# Evidence of a Large-Scale Functional Organization of Mammalian Chromosomes

**DOI:** 10.1371/journal.pbio.0050127

**Published:** 2007-05-15

**Authors:** Petko M Petkov, Joel H Graber, Gary A Churchill, Keith DiPetrillo, Benjamin L King, Kenneth Paigen

The recent report of Shifman et al. [1] challenged one of the findings in our report [2] supporting the concept that the mammalian genome contains blocks of functionally related genes. Among other evidence, we showed that in mice, when allelic combinations in these regions have been disrupted by genetic recombination, the resulting mice have a reduced ability to survive further inbreeding. We also suggested that this was a broader phenomenon based on an apparent genome-wide reduction in survival of recombinants among recombinant inbred lines of mice that have survived a second round of inbreeding.

The failure of Shifman et al. to confirm this latter observation derived from their inclusion of the so-called “new BXD” lines in Table 3 of their paper that served as the basis for calculating the genetic length of recombinant inbred lines. Applying the Haldane-Waddington equation to these mice for calculating genetic distances is not appropriate, because they were developed from an advanced intercross population with a resulting excess of recombinations. Examination of Table 3 confirms this. Shifman et al. have now generously made their initial data available to us; using a revised Table 3 with these lines removed and the same methodology as Shifman et al. generates a map length of 1393 ± 14 cM. This is 14.7% shorter than their estimate of 1630 cM for single meiosis genetic length of the mouse genome (their Table 1); we reported a reduction of 18.1% using different data sets and methodologies.

We have been in communication with the authors of Shifman et al., and they are in agreement that this new formulation of the results is correct.

We conclude that the recalculated results of Shifman et al. now provide additional support for our original conclusions regarding the functional organization of mammalian genomes.

We are grateful to Dr. Karl Broman of Johns Hopkins University for his invaluable assistance with the calculations.
